# Case report: Successful treatment of an anti-D2R and DPPX antibody-associated autoimmune encephalitis patient with high-dose methylprednisolone and intravenous immunoglobulin

**DOI:** 10.3389/fimmu.2024.1338714

**Published:** 2024-02-26

**Authors:** Zhangliang Lin, Feng Zhou, Lili Ni, Shiye Dong, Guoping Fu, Jiangman Zhao

**Affiliations:** ^1^ Neurology Department, Shaoxing No.2 Hospital Meical Community General Hospital, Shaoxing, China; ^2^ Department of Medicine, Shanghai Biotecan Pharmaceuticals Co., Ltd., Shanghai Zhangjiang Institute of Medical Innovation, Shanghai, China

**Keywords:** autoimmune encephalitis, anti-D2R antibody, anti-DPPX antibody, epilepsy, methylprednisolone, intravenous immunoglobulin

## Abstract

**Background:**

Autoimmune encephalitis is a neurological condition caused by abnormal immune responses, manifesting as cognitive impairments, behavioral abnormalities, and seizures. Its diagnosis depends on the detecting neuronal surface antibodies in serum or cerebrospinal fluid. Despite recent advances in understanding, clinical recognition remains challenging, especially with rare antibodies such as anti-dopamine D2 receptor (D2R) and anti-dipeptidyl-peptidase-like protein 6 (DPPX) antibodies. Delayed diagnosis can lead to severe complications. This case presentation emphasizes the diagnostic intricacies and effective treatment of the anti-D2R and DPPX antibody-associated autoimmune encephalitis.

**Case description:**

The patient presented with a 3-day history of fatigue and limb soreness followed by a 3-h episode of confusion and limb convulsions. Upon admission to our facility, the initial diagnosis included status epilepticus, aspiration pneumonia, metabolic acidosis, respiratory alkalosis, and suspected encephalitis. Despite receiving antiepileptic, anti-infection, and antivirus therapy, the patient’s condition deteriorated. Both computed tomography (CT) scan and magnetic resonance imaging (MRI) of the brain showed no significant abnormalities. No pathogen was identified in the cerebrospinal fluid (CSF). However, further CSF and serum examination revealed positive results of anti-D2R and anti-DPPX antibodies, confirming a diagnosis of anti-D2R and DPPX antibody-associated autoimmune encephalitis. The patient underwent a comprehensive treatment regimen, including high-dose methylprednisolone pulse therapy combined with intravenous immunoglobulin (IVIG), antiviral and anti-infection treatments, and antiepileptic medications. Significant clinical improvement was observed, and by the 18th day of admission, the patient was stable and coherent.

**Conclusions:**

The current patient represents the first reported case of double-positive autoimmune encephalitis for anti-D2R and DPPX antibodies, with epilepsy as a prominent feature. High-dose methylprednisolone pulse therapy combined with IVIG has shown significant safety and efficacy in treating anti-D2R and DPPX antibody-positive autoimmune encephalitis-associated epilepsy.

## 1 Introduction

Autoimmune encephalitis is an intricate neurological disorder characterized by inflammation of the brain due to an immune system attack on neuronal cells (1). In clinical practice, the underdiagnosis of autoimmune encephalitis poses a significant challenge. The disease manifests with a diverse range of symptoms, including cognitive impairment, behavioral abnormalities, motor dysfunction, and seizures, leading to overlaps with other neurological disorders (2). The lack of specificity in these symptoms makes it susceptible to misdiagnosis during the initial assessment (2, 3). Furthermore, the exact etiology and course of autoimmune encephalitis remain incompletely understood, adding to the complexity of its diagnosis. The delay in diagnosis can result in missed opportunities for early intervention, with immunotherapy playing a pivotal role in the management of autoimmune encephalitis (4). Therefore, it is imperative to enhance medical awareness of autoimmune encephalitis, intensify vigilance for early detection, and further improve diagnostic tools and methods in clinical settings.

The detection of specific autoantibodies has emerged as a crucial tool in diagnosing autoimmune encephalitis, providing valuable insights into the underlying immunological mechanisms, and guiding targeted therapeutic interventions (5). Identifying autoantibodies associated with autoimmune encephalitis, such as anti-N-methyl-D-aspartate receptor (NMDAR) antibodies and anti-leucine-rich glioma-inactivated 1(LGI1) antibodies, has significantly contributed to refining the diagnostic process. These antibodies serve as specific biomarkers, helping clinicians distinguish autoimmune encephalitis from other neurological conditions with similar clinical presentations. Furthermore, the presence of certain autoantibodies often correlates with distinct clinical phenotypes and outcomes. For instance, the detection of anti-NMDAR antibodies is frequently associated with psychiatric symptoms (6), whereas anti-LGI1 antibodies are linked to limbic encephalitis (7). This correlation enhances the specificity of the diagnosis and facilitates personalized treatment strategies tailored to the patient’s antibody profile.

Autoimmune encephalitis associated with anti-dopamine D2 receptor (D2R) and anti-dipeptidyl-peptidase-like protein 6 (DPPX) antibodies is relatively rare (8, 9), but it is observed in certain populations, with a higher prevalence in younger individuals (8). Due to the relative rarity of these cases, in-depth studies are needed to improve the diagnosis and treatment of such conditions. In this case report, we present an anti-D2R and DPPX antibody-associated autoimmune encephalitis patient, whose primary clinical manifestation was epilepsy. This case report provides valuable insights for clinicians in the diagnosis of autoimmune encephalitis with coexistence of different antibodies. It helps expand understanding of the subtypes of patients with autoimmune encephalitis. The successful treatment of this patient using a regimen of methylprednisolone and intravenous immunoglobulin offers clinicians an effective therapeutic approach, particularly for those with similar antibody profiles.

## 2 Case description

A 53-year-old Han Chinese man was admitted to our emergency department with a 3-day history of fatigue and limb soreness. This was followed by an episode of limb convulsions lasting 4 min–5 min, resulting in a loss of consciousness lasting 3 h, accompanied by urinary and fecal incontinence. The patient reported no fever, chills, cough and sputum, chest distress, or shortness of breath. The patient’s family noted his generally good health, without previous medical records of internal medicine conditions such as hypertension, diabetes, heart disease, or kidney disease. Additionally, there was no record of infectious diseases like tuberculosis or hepatitis, major surgeries, injuries, blood transfusions, poisonings, current medications use, substance addiction, or known allergies to medications or foods. The patient has a regular alcohol and smoking habit for over 30 years, but no prolonged exposure to industrial dust, toxic substances, or radioactive materials. Furthermore, known autoimmune diseases, brain viral infections, epilepsy, or related conditions were ruled out. The patient denied any hereditary diseases in both parental lineages for three generations.

**Figure 1:**
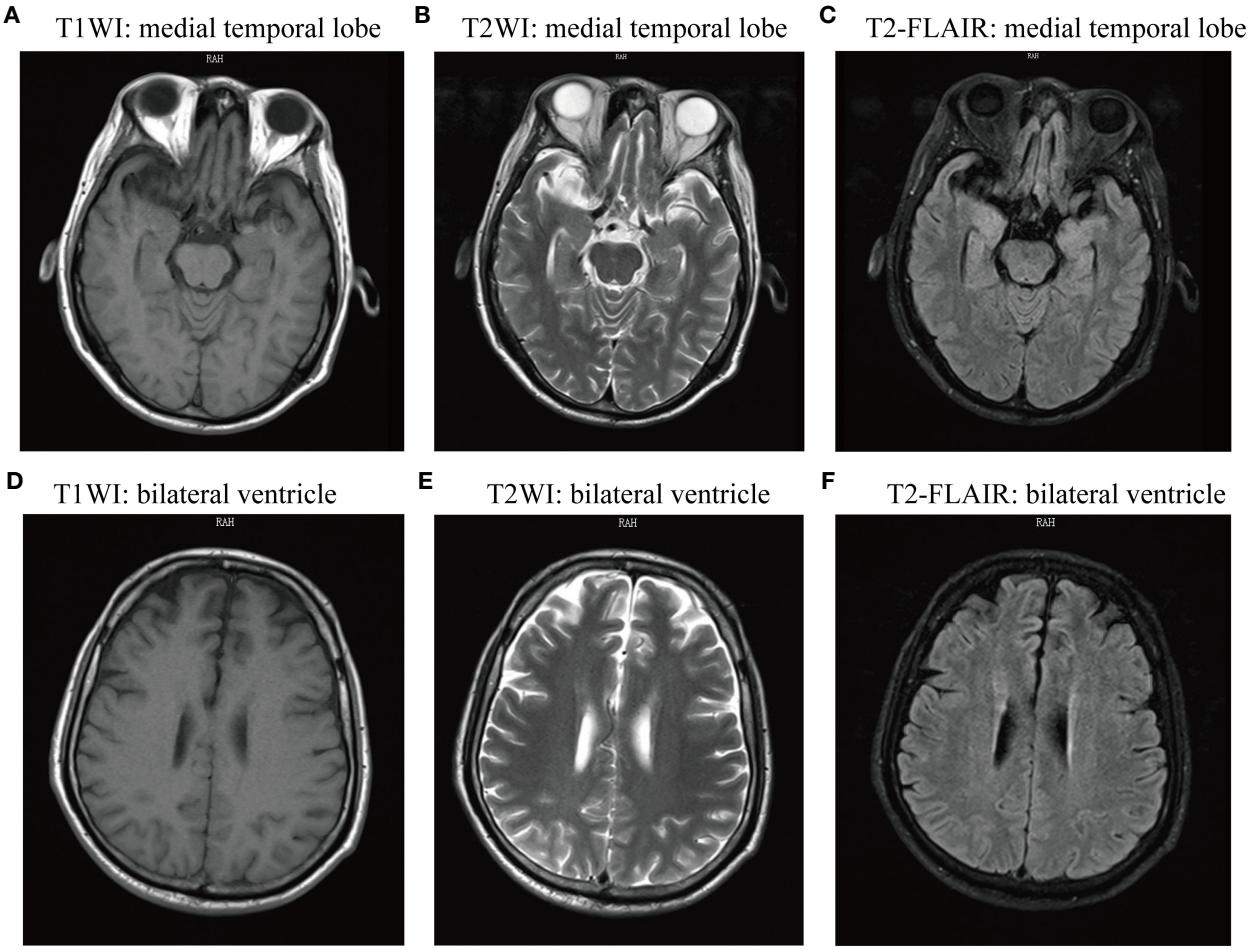
Magnetic resonance imaging (MRI) of the brain showed no abnormal signs. The two sides of the brain were symmetric, the gray matter and white matter were clearly demarcated, and there was no obvious abnormal signal shadow on T1WI, T2WI, FLAIR, and DWI axial view. **(A-C)** T1WI, T2WI, and T2-FLAIR axial view presenting medial temporal lobe; **(D-E)** T1WI, T2WI, and T2-FLAIR axial view presenting lateral ventricle. T1WI, T1-weighted imaging; T2WI, T2-weighted imaging; T2-FLAIR, T2-weighted fluid attenuated inversion recovery.

During the initial physical examination, the patient presented with a temperature of 36.8 and loss of consciousness. His pupils were equal in size, round, and 3 mm in diameter, with a responsive light reflex. Bilateral frontal wrinkles were symmetrical, and neck resistance was evident. While the muscle tone in all four limbs appeared normal, the patient was uncooperative during the muscle strength examination. The Hoffman’s sign showed negative results, as did the Kernig’s sign. The Babinski sign results showed to be positive on the left side and suspiciously positive on the right side. Abnormal laboratory findings included a decreased lymphocyte percentage at 0.16 (normal range: 0.20–0.50), elevated blood glucose at 7.44 mmol/L (normal range: 3.89 mmol/L–6.11 mmol/L), increased creatinine at 149 μmol/L (normal range: 57 μmol/L–97 μmol/L), and hypokalemia at 2.96 mmol/L (normal range: 3.4 mmol/L–4.5 mmol/L). Both CT scan and MRI of the brain revealed no abnormalities (**Figure 1**). Upon admission to the Neurology Department, the preliminary diagnosis included status epilepticus, aspiration pneumonia, metabolic acidosis, respiratory alkalosis, and suspected encephalitis. For antiepileptic treatment, sodium valproate was administered at a dose of 0.8 g once *via* microinfusion pump, and diazepam at 10 mg once *via* intravenous injection. The patient also received intravenous ceftriaxone 2.0 g once daily for anti-infective treatment and intravenous acyclovir at 0.5 g every 8 h for antiviral treatment. Unfortunately, the patient’s condition deteriorated, with fluctuating oxygen saturation (oscillating around 92%) and persistent episodes of convulsions accompanied by vomiting. Due to the continuous threat of cardiac and respiratory arrest, the patient was transferred to the Intensive Care Unit (ICU). In the ICU, mechanical ventilation therapy was initiated to stabilize oxygen saturation levels. A critically ill notice was issued for the patient.

**Figure 2:**
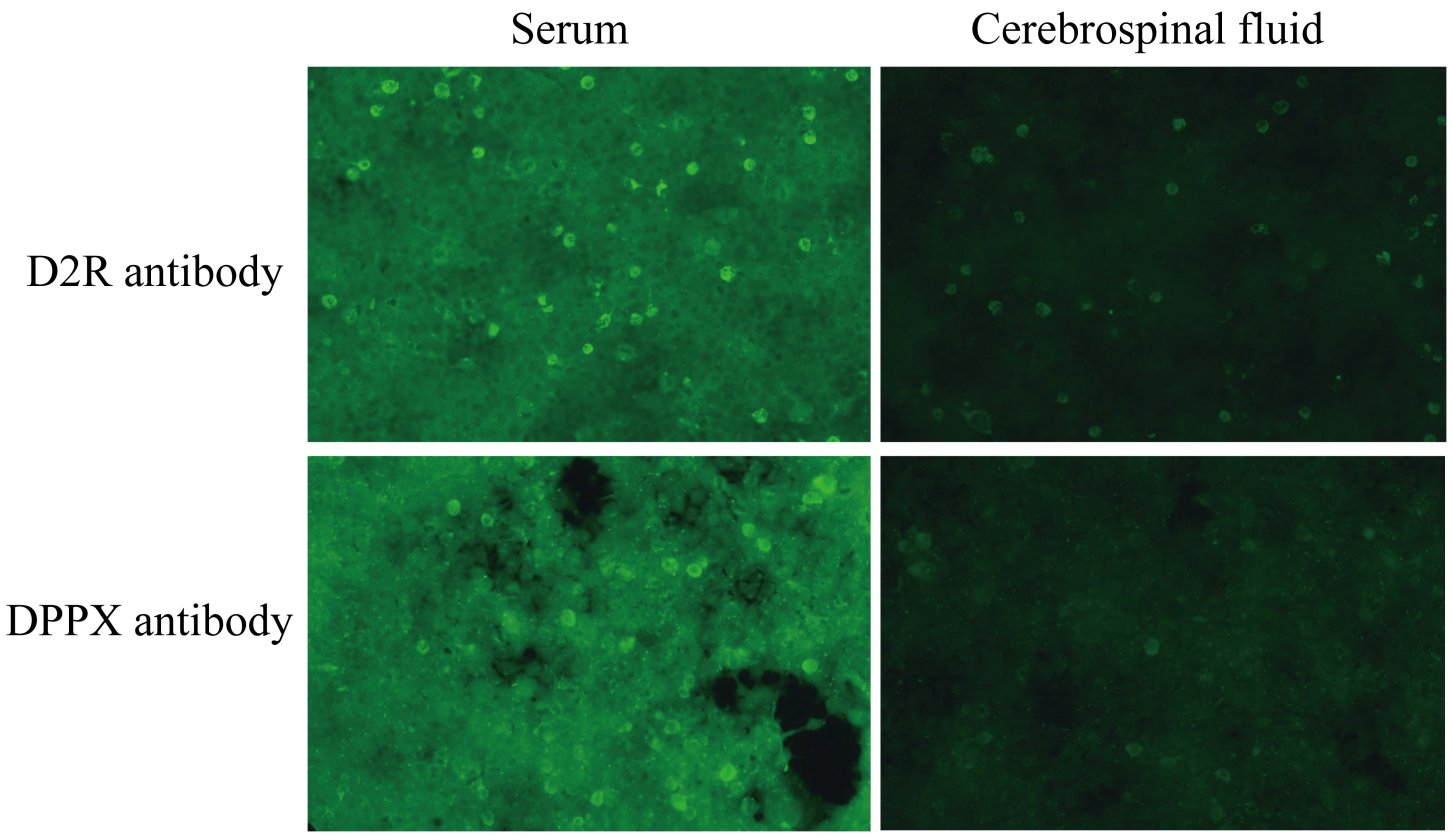
The results of autoimmune encephalitis antibody examination in cerebrospinal fluid and serum by indirect immunofluorescence cell-based assay (CBA-IIF).

On the second day of admission, the patient remained unconscious with persistent status epilepticus. Antiepileptic and anti-infective treatments were ongoing. A lumbar puncture was conducted for cerebrospinal fluid (CSF) examination, encompassing biochemistry tests, pathogen culture, acid-fast staining, and T-SPOT.TB test. No pathogen was identified in CSF, and the data of CSF biochemistry and protein tests were within the normal range. CSF and serum samples were collected for examination of autoimmune encephalitis antibodies, including anti-NMDAR1a, anti-AMPAR1, anti-AMPAR2, anti-LGI1, anti-CASPR2, anti-GABABR, anti-DPPX, anti-IgLON5, anti-GlyR1, anti-GABAARα1, anti-GABAARβ3, anti-γ2, anti-mGluR5, anti-D2R, anti-Neurexin3α, anti-KCNA4, anti-NMDAR2a, anti-NMDAR2b antibody IgG. The significant findings included a positive anti-D2R antibody in the CSF and positive anti-D2R and anti-DPPX antibodies in the serum (**Figure 2**). By the third day of admission, based on the clinical manifestations, epidemiological features, laboratory results, and brain CT/MRI imaging, the patient was definitively diagnosed with anti-D2R and anti-DPPX antibody-associated autoimmune encephalitis.

The treatment plan for the patient involved high-dose methylprednisolone pulse therapy combined with IVIG. Specifically, methylprednisolone was administered at a dose of 1,000 mg per day for pulse therapy over 3 days, followed by a reduced dose of 500 mg per day for an additional 3 days. Subsequently, maintenance treatment consisted of intravenous infusion at 80 mg per day. Simultaneously, IVIG was administered at a dose of 25 g per day for supportive treatment. Given elevated infection markers and concurrent aspiration pneumonia, anti-infective therapy with piperacillin–tazobactam and antiviral treatment with acyclovir were continued. Seizure management remained a critical part of the ongoing care. The patient’s condition was closely monitored for any changes, with prompt symptomatic interventions as needed to maintain internal homeostasis and stability.

**Figure 3:**
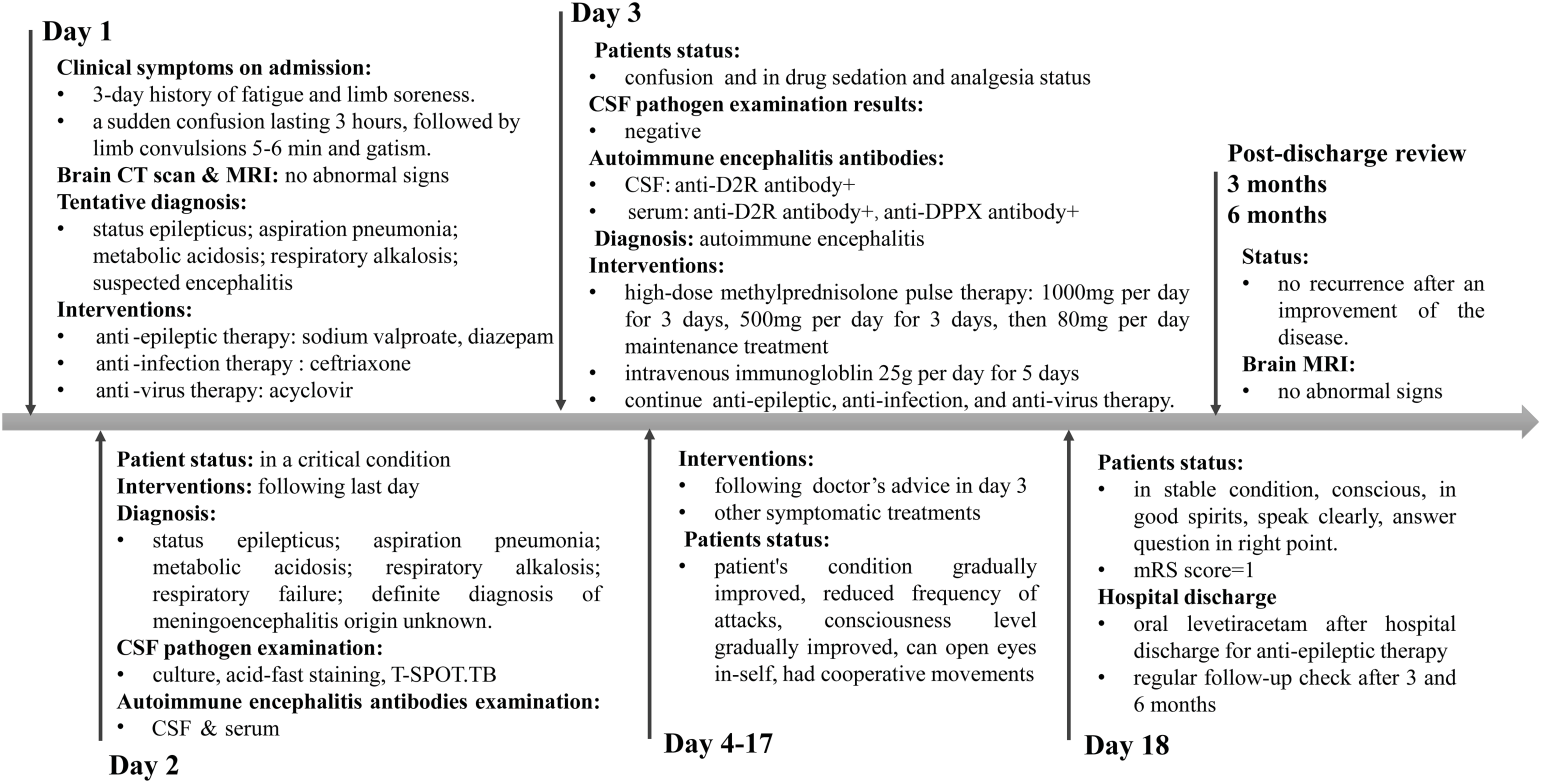
Timeline of the patient’s diagnosis and treatment process. Day 1 means the day of admission to emergency department.

During the treatment course from days 4 to 17, the steroid dosage was gradually reduced. The patient exhibited notable improvement, evidenced by a reduced frequency of seizures. There was a gradual return to clarity of consciousness, with the patient regaining the ability to open his eyes and exhibiting coordinated movements. On the 18th day of admission, the patient’s condition was stable, with clear consciousness, alert mental state, articulate speech, and appropriate responses. The treatment with levetiracetam tablets for antiepileptic therapy was continued. Upon discharge, the patient’s condition was assessed with a Modified Rankin Scale (mRS) score of 1, indicating minimal disability. The patient continued to take levetiracetam tablets orally post-discharge. Follow-up examinations at 3 and 6 months indicated improvement in the patient’s condition with no recurrence. The timeline of the patient’s diagnosis and treatment process is presented in **Figure 3**.

## 3 Discussion

Autoimmune encephalitis, a non-infectious cause of encephalitis, rarely involves antibodies against D2R and DPPX (9). Autoimmune encephalitis with anti-D2R antibodies tends to affect a younger demographic, with a predilection for adolescents and young adults (8). The pathogenic mechanisms underlying the development of anti-D2R antibodies in autoimmune encephalitis remain an area of active investigation. Current hypotheses suggest an aberrant immune response targeting dopamine receptors, leading to neuroinflammation and subsequent neurological symptoms (10). Addabbo et al. found an association between circulating anti-D2R autoantibodies and the exacerbation of tics in children with chronic tic disorders (11). Anti-D2R autoantibodies were reported to be related with movement disorders such as Sydenham’s chorea (10, 12). Autoimmune encephalitis caused by anti-DPPX autoantibody is an infrequent occurrence, typically characterized by a triad of symptoms—weight loss, central nervous system hyperactivity, and cognitive deficits. Recent reports suggested a more diverse clinical presentation (13). Xiao et al.’s study (14) expanded clinical phenotypes of anti-DPPX encephalitis, including prodromal fever, diarrhea, sleep disturbances with abnormal sleep behavior featuring rapid eye movements, limb paralysis, and marked pleocytosis.

To our knowledge, the present patient represents the first reported case of double-positive encephalitis associated with anti-D2R and anti-DPPX antibodies. The prodromal symptoms included fatigue and limb soreness followed by rapidly progressive epileptic seizure. Brain CT scan and MRI showed no abnormal signs. Unfortunately, autoimmune encephalitis diagnosis was overlooked during the initial assessment on the first day of admission. Symptomatic treatments, including antiepileptic, anti-infective, and antiviral therapy, worsened the patient’s condition. Indirect immunofluorescence cell-based assay (CBA-IIF) identified positive anti-D2R and anti-DPPX antibodies in serum and weakly positive anti-D2R antibody in CSF, providing a clear diagnosis of anti-D2R and anti-DPPX antibody-associated autoimmune encephalitis. This case highlights that status epilepticus was the prominent feature of anti-D2R and anti-DPPX antibody-associated autoimmune encephalitis. Both anti-D2R and anti-DPPX antibodies targeting dopamine receptors and potassium channels, respectively, may contribute to neuroinflammation, leading to dysfunction in neurotransmission and neuronal activity, contributing to prolonged and severe seizures. CBA-IIF has become an instrumental technique in autoimmune encephalitis diagnostics; rare neuronal surface antibodies should not be overlooked in the initial test when encountering suspected autoimmune encephalitis or epileptic patients.

Immunotherapy has emerged as a crucial component in the management of autoimmune encephalitis, demonstrating notable efficacy in mitigating symptoms and improving outcomes for affected individuals (4). The primary goal of immunotherapy is to modulate the aberrant immune response responsible for attacking neural tissues, thereby reducing inflammation and preventing further damage. Various immunotherapeutic approaches have been employed, including corticosteroids, IVIG, plasma exchange (PLEX), and more targeted agents such as rituximab (4, 15). Corticosteroids, like methylprednisolone, are often used as initial treatments to rapidly suppress inflammation (16). IVIG and PLEX aim to modulate the immune system by providing immune-regulatory factors or removing pathogenic antibodies, respectively (17, 18). While responses to treatment can vary among individuals, many patients experience significant improvements in symptoms and quality of life. However, the optimal timing, duration, and combination of immunotherapeutic agents remain subjects of ongoing research, emphasizing the need for personalized treatment strategies based on individual patient profiles and disease characteristics. In the present case report, on the third day of admission, this patient received high-dose methylprednisolone pulse therapy combined with IVIG. There is scarcity of clinical evidence regarding the efficacy and safety of the combined use of IVIG and high-dose corticosteroids (19). In the presented case, the patient demonstrated a favorable outcome following the implementation of this combined therapeutic approach.

In summary, the present patient represents the first reported case of anti-D2R and anti-DPPX antibody double-positive encephalitis, with epilepsy as a prominent feature. High-dose methylprednisolone pulse therapy combined with IVIG has shown significant safety and efficacy in epilepsy associated with anti-D2R and DPPX antibody-positive autoimmune encephalitis. The application of autoimmune encephalitis-specific antibody testing has proven invaluable in enhancing diagnostic accuracy and guiding appropriate therapeutic interventions. The identification of distinct autoantibodies not only aids in differentiating autoimmune encephalitis from other neurological disorders but also provides valuable prognostic information, ultimately contributing to more effective patient management.

## Data availability statement

The original contributions presented in the study are included in the article/supplementary material. Further inquiries can be directed to the corresponding authors.

## Ethics statement

The studies involving humans were approved by The Ethics Committee of the Shaoxing No.2 Hospital Meical Community General Hospital. The studies were conducted in accordance with the local legislation and institutional requirements. The participants provided their written informed consent to participate in this study. Written informed consent was obtained from the individual(s) for the publication of any potentially identifiable images or data included in this article. Written informed consent was obtained from the participant/patient(s) for the publication of this case report.

## Author contributions

ZL: Data curation, Writing – original draft, Writing – review & editing. FZ: Data curation, Writing – review & editing. LN: Data curation, Writing – review & editing. SD: Data curation, Writing – review & editing. GF: Data curation, Writing – original draft, Writing – review & editing. JZ: Writing – original draft, Writing – review & editing.
